# Association of plant-based dietary patterns with the risk of type 2 diabetes mellitus using cross-sectional results from RaNCD cohort

**DOI:** 10.1038/s41598-024-52946-z

**Published:** 2024-02-15

**Authors:** Neda Heidarzadeh-Esfahani, Mitra Darbandi, Firoozeh Khamoushi, Farid Najafi, Davood Soleimani, Mozhgan Moradi, Ebrahim Shakiba, Yahya Pasdar

**Affiliations:** 1 Department of Nutritional Sciences, School of Nutritional Sciences and Food Technology, Kermanshah University of Medical Sciences, Kermanshah, Iran; 2 Ala Cancer Control and Prevention Centre, Isfahan, Iran; 3 Research Center for Environmental Determinants of Health (RCEDH), Health Institute, Kermanshah University of Medical Sciences, Kermanshah, Iran; 4 Cardiovascular Research Center, Kermanshah University of Medical Sciences, Kermanshah, Iran; 5 Internal Medicine Department, School of Medicine, Kermanshah University of Medical Sciences, Kermanshah, Iran; 6 Social Development and Health Promotion Research Center, Kermanshah University of Medical Sciences, Kermanshah, Iran

**Keywords:** Diseases, Endocrinology, Health care, Medical research

## Abstract

The prevalence of type 2 diabetes mellitus (T2DM) is increasing in middle- and low-income countries, and this disease is a burden on public health systems. Notably, dietary components are crucial regulatory factors in T2DM. Plant-based dietary patterns and certain food groups, such as whole grains, legumes, nuts, vegetables, and fruits, are inversely correlated with diabetes incidence. We conducted the present study to determine the association between adherence to a plant-based diet and the risk of diabetes among adults. We conducted a cross-sectional, population-based RaNCD cohort study involving 3401 men and 3699 women. The plant-based diet index (PDI) was developed using a 118-item food frequency questionnaire (FFQ). Logistic regression models were used to evaluate the association between the PDI score and the risk of T2DM. A total of 7100 participants with a mean age of 45.96 ± 7.78 years were analysed. The mean PDI scores in the first, second, and third tertiles (T) were 47.13 ± 3.41, 54.44 ± 1.69, and 61.57 ± 3.24, respectively. A lower PDI was significantly correlated with a greater incidence of T2DM (T1 = 7.50%, T2 = 4.85%, T3 = 4.63%; P value < 0.001). Higher PDI scores were associated with significantly increased intakes of fibre, vegetables, fruits, olives, olive oil, legumes, soy products, tea/coffee, whole grains, nuts, vitamin E, vitamin C, and omega-6 fatty acids (P value < 0.001). After adjusting for confounding variables, the odds of having T2DM were significantly lower (by 30%) at T3 of the PDI than at T1 (OR = 0.70; 95% CI = 0.51, 0.96; P value < 0.001). Our data suggest that adhering to plant-based diets comprising whole grains, fruits, vegetables, nuts, legumes, vegetable oils, and tea/coffee can be recommended today to reduce the risk of T2DM.

## Introduction

Type 2 diabetes mellitus (T2DM) is a significant healthcare challenge worldwide and contributes to more than 1 million deaths annually as a primary cause of mortality ^1^. The global incidence of T2DM continues to increase, with more than 500 million people affected by T2DM by 2023^2^. Implementing preventive strategies involving modifiable lifestyle factors such as diet, smoking status, stress, and physical activity contributes significantly to reducing the economic and clinical burden of T2DM ^3,4^. Substantial evidence supports the idea that adherence to various healthy dietary patterns, including Mediterranean-style diets, Dietary Approaches to Stop Hypertension (DASH) diets, prudent patterns, and dietary guidelines, plays a crucial role in reducing susceptibility to type 2 diabetes^5^. A plant-based diet is characterized by the consumption of vegetables, fruits, nuts, legumes, seeds, and whole grains, particularly excluding certain animal products ^6^. Dietary patterns rich in plant-derived foods and low in animal-derived foods are associated with a lower incidence of chronic diseases, as indicated in the 2015 DGAC Scientific Report ^7^. Emerging evidence suggests that plant-based dietary patterns are linked to a reduced risk of cardiovascular diseases (CVDs) and dyslipidemia ^8,9^, gastroesophageal disease ^10^, cancer ^11^, gestational diabetes mellitus (GDM) ^12^, T2DM ^6^, and obesity ^13,14^. Moreover, a plant-based diet is likely to ameliorate common complications in diabetic patients, namely, sleep disturbances and psychological disorders^15^.

Epidemiologic studies have demonstrated the protective effects of certain plant-derived substances, such as polyphenols comprising flavonoid compounds, phenolic acids, stilbenes, and lignans, on insulin sensitivity to prevent T2DM by combining data from 18 cohorts^16,17^. Other potential strategies for the antidiabetic effect of a plant-based diet include reducing systemic inflammation and oxidative stress, enhancing the gut microbiota, and promoting weight loss ^6,18^. According to a recent meta-analysis of observational studies, plant-based diets are inversely associated with T2DM in high-income countries, while this correlation remains unknown in low- or middle-income countries^6^. Dietary patterns and composition (i.e., consumption of grains) varied between low- and high-income countries, which is why the results may not be generalizable to all races^19,20^.

To the best of our knowledge, in Iran, no studies have investigated the association between a plant-based diet and the risk of T2DM. In addition, the results of other aspects of research in Iran, such as CVD incidence, GDM incidence and cancer risk, are controversial ^21–23^. Therefore, in the present study, we sought to determine whether a plant-based diet can moderate the likelihood of T2DM in low- and middle-income countries to become a crucial public health priority.

## Methods

### Study design and participants

**Figure 1:**
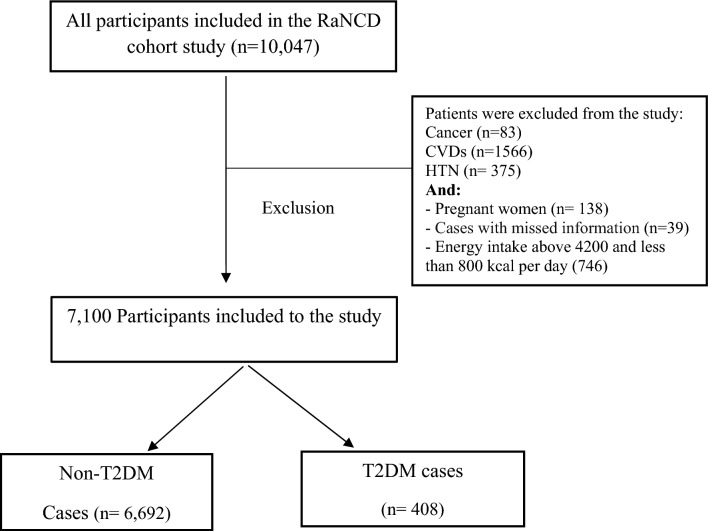
Flow chart of the study.

This cross-sectional study utilized data obtained from the Ravansar Non-Communicable Disease (RaNCD) cohort and was conducted with the approval of Kermanshah University of Medical Sciences (grant number: 92472). The RaNCD is an ongoing community-based prospective study aimed at assessing noncommunicable diseases within the Kurdish population. Comprehensive details of this study have been previously published ^24^. Initially, 10,047 participants were enrolled in the study during the baseline phase of the RaNCD study. Participants were excluded based on specific criteria: had an energy intake above 4200 or less than 800 kcal per day (746), had cancer (83), had a cardiovascular disease (1566), had hypertension (375), or were pregnant (138). After applying the exclusion criteria, a total of 7100 participants were included in this analysis (see Fig. 1).

### General measurements

Information regarding age, sex, marital status, and smoking habits was gathered through trained interviewers and online questionnaires. Physical activity levels were assessed using the PERSIAN cohort questionnaire and categorized into three groups: low (24–36.5 MET/hour per day), moderate (36.6–44.4 MET/hour per day), and high (≥ 44.5 MET/hour per day)^25,26^. Socioeconomic status (SES) was determined using variables related to residence, level of literacy, and economic well-being and was classified into three groups ranging from lowest to highest levels ^24^.

Anthropometric data, including weight, visceral fat area (VFA), and body mass index (BMI), were obtained using an automated bioelectric impedance device (In Body Co., Seoul, Korea). Additionally, waist circumference (WC) was measured to the nearest 0.1 cm. WC was measured at the narrowest point immediately below the lowest rib and above the iliac crest ^24^.

### Dietary assessment and plant-based diet index (PDI) calculation

A trained dietitian collected dietary data using a validated semiquantitative food frequency questionnaire (FFQ) consisting of 118 food items. This FFQ was utilized for the annual assessment of typical food and beverage intake.

The FFQ estimates the intake frequency (per day, week, month, and year) and quantity of each food item consumed. Food items were categorized into 18 groups based on their nutrient content, as demonstrated in Table 1. These groups were further classified into broader categories: healthy plant foods, less healthy plant foods, and animal foods. Healthy plant foods included whole grains, fruits, vegetables, nuts, legumes, vegetable oils, tea, and coffee, while less healthy foods included fruit juices, refined grains, potatoes, sugar-sweetened beverages, and sweets/desserts.

**Table 1:** The distribution of food items within each food group.

Plant food groups	
Healthy	
Whole grains	Dark breads (e.g., barbari, sangak, taftun), Baked barley/oats, bran breads, others
Fruits	Melon, watermelon, honeydew melon, plums, prunes, apples, cherries, sour cherries, peaches, nectarine, pear, fig, date, grapes, kiwi, pomegranate, strawberry, banana, persimmon, berry, pineapple, oranges, dried fruits, olive, others
Vegetables	Cauliflower, carrot, tomato and its products, spinach, lettuce, cucumber, eggplant, onion, greens, green bean, green pea, squash, mushroom, pepper, corn, garlic, turnip, others
Nuts	Almonds, peanut, walnut, pistachio, hazelnut, seeds, others
Legumes	Lentils, split pea, beans, chick pea, fava bean, soy, others
Vegetable oils	Olive oil, oil-based salad dressing, vegetable oil used for cooking
Tea and Coffee	Tea, coffee, decaffeinated coffee
Less healthy	
Fruit juices	Apple juice, orange juice, grapefruit juice, other fruit juice
Refined grains	Lavash bread, baguette bread, rice, pasta, biscuits, crackers, pasta, others
Potatoes	French fries, baked or mashed potatoes, potato or corn chips
Sugar sweetened beverages	Soft drinks, sugar sweetened beverages
Sweets and Desserts	Cookies, cakes, biscuits, muffins, pies, chocolates, honey, jam, sugar cubes, sweet tahini, sugar, candies, others
Animal food groups	
Animal fats	Butter added to food, butter or lard used for cooking
Dairies	Low-fat milk, skim milk, low-fat yogurt, cheese, curd, yogurt drink, High-fat milk, high-fat yogurt, cream cheese, cream, dairy fat, ice cream, others
Eggs	Eggs
Fish or Seafood	Canned tuna, all other fishes
Meat	Beef and veal, lamb, minced meat, sausage, deli meat, hamburger, chicken, heart, kidney, liver, tongue, brain, offal, rennet
Misc. animal-based foods	Pizza, chowder or cream soup, mayonnaise

Furthermore, animal foods consisted of animal fats, eggs, dairy products, fish/seafood, meats, and other animal-based items. Each participant received a score ranging from 1 to 5 for every food group based on their quintile of consumption.

To compute the plant-based diet index (PDI), the highest quintile within healthy plant foods and less healthy plant foods were allotted 5 points, mirroring the highest scores as similar to the lowest quintile within animal foods. Conversely, the lowest quintile of healthy plant foods and less healthy plant foods, as well as the highest quintile of animal foods, received the lowest score (1 point). The cumulative scores of all food groups were then calculated to derive the overall index scores, ranging theoretically from 18 to 90 and representing the lowest to the highest possible scores ^27^.

### Definition of type 2 diabetes mellitus

For the diagnosis of T2DM, fasting blood sugar (FBS) levels were evaluated in accordance with the International Diabetes Federation (IDF) criteria, which consider levels equal to or greater than 126 mg/dL and/or treatment with antidiabetic medications ^28^.

### Statistical analysis

Statistical analyses were performed using Stata version 14.2 software (Stata Corp., College Station, TX, USA). The Kolmogorov–Smirnov test was used to test the normality of the variables. Differences in continuous variables across the PDI tertiles were assessed using analysis of variance (one-way ANOVA), while categorical variables were compared using the chi-square test. The means ± SDs of qualitative characteristics across the different tertiles of adherence to the PDI were calculated. All dietary intakes of participants across tertiles of plant-based diet adjusted for daily energy intake.

Logistic regression models were employed to evaluate the associations between the PDI score and T2DM incidence. Two different models were used in this analysis: (1) Model 1, adjusted for age and sex; and (2) Model 2, adjusted for age, sex, energy intake, physical activity and SES. We selected the covariates according to previous studies ^29–32^. A P value of < 0.05 with 95% confidence intervals (CIs) was considered to indicate statistical significance in this study.

### Ethics approval and consent to participate

The study was approved by the ethics committee of Kermanshah University of Medical Sciences (KUMS.REC.1394.318). All methods were carried out in accordance with relevant guidelines and regulations. All the participants provided oral and written informed consent.

## Results

### Basic participant characteristics

Baseline information on the participants is presented across the tertiles of PDI scores in Table 2. A total of 7100 participants, with a mean age of 45.96 years, BMI of 96.43 cm, and visceral fat area of 118.07 kg, were included in the current survey. Of these participants, 47.90% were male and 52.10% were female, with 408 individuals (5.74%) diagnosed with T2DM. The mean PDI scores in the first, second, and third tertiles (T) were 47.13 ± 3.41, 54.44 ± 1.69, and 61.57 ± 3.24, respectively.

**Table 2:** Characteristics of study participants across the tertiles (T) of the plant-based diet index (PDI).

Variables	Total (n = 7100)	Plant-based diet index			P value*
		T1 (n = 2574)	T2 (n = 2516)	T3 (n = 2010)	
		Mean ± SD/Frequency (Percent)			
Plant-based diet index	53.81 ± 6.45	47.13 ± 3.41	54.44 ± 1.69	61.57 ± 3.24	–
Age (year)	45.96 ± 7.78	46.66 ± 8.09	45.61 ± 7.67	45.47 ± 741	< 0.001
Gender					
Male	3401 (47.90)	981(28.84)	1266 (37.22)	1154 (33.93)	< 0.001
Female	3699 (52.10)	1593 (43.07)	1250 (33.79)	856 (23.14)	
Marital status					
Married	6376 (89.80)	2218 (86.17)	2290 (91.02)	1868 (92.94)	< 0.001
Single	371 (5.23)	193 (7.50)	111(4.41)	67 (3.33)	
Widowed/Divorced	353 (4.97)	163 (6.33)	115 (4.57)	75 (3.73)	
Socio-economic status					
1 (lowest)	2257 (31.81)	1000 (38.87)	759 (30.20)	498 (24.78)	< 0.001
2	2359 (33.24)	837 (32.53)	796 (31.68)	726 (36.12)	
3 (Highest)	2480 (34.95)	736 (28.60)	958 (38.12)	786 (39.10)	
Physical activity (Met-h/day)					
Light	2092 (29.46)	732 (28.44)	741(29.45)	619 (30.80)	0.001
Moderate	3432 (48.34)	1321(51.32)	1207(47.97)	904 (44.98)	
High	1576 (22.20)	521(20.24)	568 (22.58)	487 (24.23)	
T2DM	408 (5.75)	193 (7.50)	122 (4.85)	93 (4.63)	< 0.001
Anti-T2DM medications	231 (11.64)	126 (15.22)	65 (9.66)	40 (8.28)	< 0.001
Current Smoker	834 (11.82)	246 (9.59)	303 (12.11)	285 (4.33)	< 0.001
Body mass index (kg/m^2^)	27.13 ± 4.57	26.91 ± 4.48	27.12 ± 4.59	27.43 ± 4.63	< 0.001
Waist circumference (cm)	96.43 ± 10.34	96.17 ± 10.23	96.39 ± 10.38	96.81 ± 10.40	0.113
Visceral fat area (cm^2^)	118.07 ± 50.62	119.54 ± 49.66	116.81 ± 50.52	117.75 ± 51.93	0.151
Fasting blood sugar (mg/dl)	94.62 ± 26.17	96.29 ± 30.13	93.64 ± 22.78	93.68 ± 24.50	< 0.001

*P-value was obtained t-test Chi-square test.

Participants with higher PDI scores tended to have a higher socioeconomic status, were more likely to be married, exhibited lower levels of physical activity, and had a reduced risk of current smoking (P < 0.001). Additionally, a significant association was observed between lower PDI scores and a greater incidence of T2DM (T1 = 7.50%, T2 = 4.85%, T3 = 4.63%; P < 0.001) (Table 2).

### PDI and dietary intake

In Table 3, the mean dietary intakes of participants across different tertiles of PDI are depicted, with energy intake adjusted. Individuals in T3 of the PDI demonstrated increased consumption of nutrients and foods, including carbohydrates, trans fats, fibre, vegetables, fruits, olives, olive oil, legumes, soy products, tea/coffee, whole grains, nuts, vitamin E, vitamin C, and omega-6 (P < 0.001).

**Table 3:** Mean and confidence interval of dietary intakes of participants across tertiles of plant-based diet index.

Food parameters	Total	Plant-based diet index			P value
		T1 (n = 2574)	T2 (n = 2516)	T3 (n = 2010)	
Energy (kcal/d)	2537.19 ± 727.58	2160.60 ± 646.05	2586.04 ± 668.03	2958.28 ± 644.40	< 0.001
Carbohydrate (%E)	61.25 ± 6.16	59.30 ± 6.28	61.57 ± 5.92	63.33 ± 5.49	< 0.001
Protein (%E)	13.72 ± 2.16	14.00 ± 2.24	13.68 ± 2.17	13.40 ± 1.99	< 0.001
Lipid (%E)	26.98 ± 5.93	28.20 ± 6.25	26.74 ± 5.78	25.71 ± 5.35	< 0.001
Saturated fat (g/d)	29.14 ± 0.19	32.70 ± 0.18	28.85 ± 0.17	24.92 ± 0.21	< 0.001
Trans fat (g/d)	0.29 ± 0.01	0.24 ± 0.01	0.28.01	0.35 ± 0.01	0.001
Seeds (g/d)	6.39 ± 0.18	6.96 ± 0.19	6.28 ± 0.18	5.82 ± 0.21	0.006
Fibre (g/d)	22.91 ± 0.011	20.63 ± 0.11	22.88 ± 0.11	25.85 ± 0.13	< 0.001
Vegetables (g/d)	472.50 ± 4.50	399.12 ± 4.81	474.36 ± 4.60	564.10 ± 5.44	< 0.001
Fruits (g/d)	274.20 ± 3.54	220.19 ± 3.85	273.14 ± 3.68	341.12 ± 4.35	< 0.001
Olive (g/d)	0.42 ± 0.03	0.28 ± 0.03	0.45 ± 0.03	0.56 ± 0.03	< 0.001
Olive oil (g/d)	0.10 ± 0.01	0.08 ± 0.01	0.10 ± 0.01	0.12 ± 0.01	0.053
Legumes (g/d)	34.15 ± 0.58	25.70 ± 0.59	33.80 ± 0.56	45.40 ± 0.67	< 0.001
Dairy (g/d)	446.19 ± 6.64	554.31 ± 6.97	437.10 ± 6.67	319.15 ± 7.88	< 0.001
Red and white meat (g/d)	72.24 ± 53.14	79.92 ± 0.98	72.82 ± 0.93	61.69 ± 1.10	< 0.001
Soy products (g/d)	3.44 ± 0.11	2.99 ± 0.11	3.40 ± 0.10	4.05 ± 0.13	< 0.001
Whole grains (g/d)	10.05 ± 0.26	7.71 ± 0.25	10.32 ± 0.24	12.80 ± 0.29	< 0.001
Egg (g/d)	21.69 ± 19.90	24.02 ± 0.39	21.61 ± 0.37	18.81 ± 0.44	< 0.001
Nuts (g/d)	8.82 ± 0.23	6.80 ± 0.21	8.83 ± 0.20	11.40 ± 0.23	< 0.001
Tea & Coffee (g/d)	722.70 ± 10.12	597.66 ± 9.90	729.84 ± 9.48	873.84 ± 11.20	< 0.001
Sweets and Desserts (g/d)	59.52 ± 0.81	48.78 ± 0.73	60.61 ± 0.69	71.91 ± 0.82	< 0.001
Vitamin E (mg/d)	7.63 ± 3.67	6.65 ± 0.10	7.66 ± 0.06	8.84 ± 0.07	< 0.001
Vitamin D (IU/d)	44.64 ± 0.60	47.99 ± 0.61	44.31 ± 0.59	40.78 ± 0.70	< 0.001
Vitamin C (mg/d)	108.95 ± 65.62	89.84 ± 1.15	109.23 ± 1.10	133.10 ± 1.30	< 0.001
Omega_3 (g/d)	0.04 ± 0.00	0.05 ± 0.00	0.04 ± 0.00	0.04 ± 0.00	< 0.001
Omega_6 (g/d)	4.65 ± 0.06	3.77 ± 0.06	4.76 ± 0.06	5.64 ± 0.07	< 0.001

The mean ± SD of food parameters is adjusted for daily energy intake.

*P-value was obtained one-way ANOVA test.

Conversely, higher adherence to the PDI was associated with lower intakes of proteins, lipids, saturated fat, seeds, dairy products, red and white meats, eggs, vitamin D, and omega-3 fatty acids (P < 0.001).

### PDI and T2DM

Table 4 displays both crude and adjusted ORs (95% CI) for T2DM across the tertiles of PDI. According to the crude model, the odds of T2DM at T3 in the DPI were significantly lower than those at T1 (OR = 0.59; 95% CI = 0.46, 0.77). This significant relationship persisted even after we adjusted for potential factors such as age and sex; a 37% decrease in the odds of developing T2DM was observed in T3 patients compared to T1 patients (OR = 0.63; 95% CI = 0.48, 0.82). Further adjustment for energy intake, physical activity, and SES revealed a 30% decrease in the odds of having T2DM at T3 compared to T1 (OR = 0.70; 95% CI = 0.51, 0.96; P-trend < 0.001).

**Table 4:** Association between the plant-based diet index and type 2 diabetes mellitus by logistic regression analysis.

	Plant-based diet index (PDI)			P value trend
	T1 (Ref)	T2 OR (95% CI)	T3 OR (95% CI)	
Crude	1	0.63 (0.50, 0.79)	0.59 (0.46, 0.77)	< 0.001
Model 1	1	0.66 (0.52, 0.84)	0.63 (0.48, 0.82)	0.005
Model 2	1	1.00 (0.79, 1.26)	0.70 (0.51, 0.96)	< 0.001

Model 1: Adjusted for age and sex.

Model 2: Adjusted for age, sex, energy intake, physical activity and SES.

**Figure 2:**
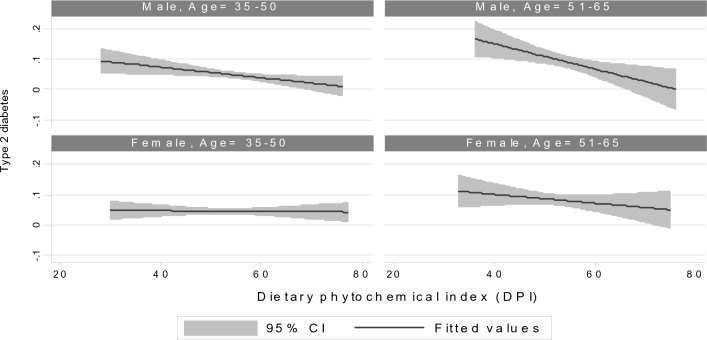
Association between the plant-based diet index and type 2 diabetes mellitus according to sex and age.

Figure 2 illustrates the association between DPI and T2DM among different sex and age groups, demonstrating inverse associations between DPI and T2DM in both sex and age categories.

## Discussion

In this study, our findings revealed that participants with higher PDI scores had lower odds of having T2DM. Notably, even after adjusting for age and sex, a significant inverse association was observed between higher PDI tertiles and a reduced risk of T2DM. This robust association persisted after we adjusted for confounding variables such as age, sex, energy intake, physical activity, and SES. Furthermore, the inverse relationship between increased energy intake from plant-rich foods and T2DM was consistent across sex and various age groups.

Prior research has extensively explored the interconnection between plant-based diets and susceptibility to T2DM. In line with our current study, Satija et al. conducted a prospective cohort study involving 200,727 individuals in the U.S. Their investigation revealed an independent association between plant-based diets, particularly high-quality plant foods, and a remarkable 49% reduction in the risk of T2DM, even after adjusting for BMI and other diabetes risk factors ^27^. Similarly, Ahmed et al. conducted a four-year prospective study involving 35,307 Swedish men and women. Their findings highlighted an elevated risk of T2DM among individuals with inadequate vegetable intake. Specifically, after accounting for BMI, age, smoking status, alcohol consumption, and physical activity, a 62% increased risk of T2DM was observed in men (OR = 1.62; 95% CI = 1.00, 2.63) ^33^. Multiple studies exploring different populations have consistently demonstrated an inverse relationship between adherence to plant-based diets and the risk of T2DM. In a cross-sectional study involving 50,694 Chinese participants, a significant association was observed between the fourth quartile of PDI and a reduced risk of T2DM (OR = 0.83; 95% CI = 0.75–0.92)^34^. Likewise, the Singapore Chinese Health Study indicated that adherence to plant-based diets and higher PDI scores correlated with a notable 17% reduction in the risk of T2DM^35^. Moreover, findings from the 7.3-year Rotterdam Study, conducted on Dutch adults by Chen et al., reported a 13% reduced risk of T2DM among individuals with higher PDI scores, even after adjusting for lifestyle confounders ^36^. Further affirming these observations, three large cohort studies conducted in the U.S. involving both men and women over a 4-year follow-up period highlighted a substantial link between better adherence to a plant-based diet and a decreased risk of T2D. Specifically, these studies reported a 12–23% increase in the risk of developing diabetes over four years among participants who exhibited the greatest decline in PDI adherence ^37^. Overall, the Henan Rural Cohort Study conducted by Yang et al. among Chinese adults revealed a notable association between a higher plant-based diet score and a reduced risk of T2DM (OR = 0.88; 95% CI: 0.79–0.98). This study highlighted a 4% decrease in the risk of developing T2DM with a one standard deviation increase in the PDI ^38^. However, it is important to note that not all studies have demonstrated a consistent relationship between T2DM incidence and PDI. For instance, the findings of two cohort studies contradicted the outcomes observed in the present study and suggested no significant relationship between T2DM incidence and PDI ^39,40^. These discrepancies might arise due to variations in populations, dietary behaviors, and lifestyles.

One such example is the study by Kim et al. in Korea, where they observed that the healthy plant-based diet index (hPDI), rather than the traditional PDI, was associated with the risk of developing T2DM. This study emphasized that in a population consuming more plant foods and fewer animal foods, solely increasing the quantity of plant foods might not be effective. However, the consumption of higher-quality plant foods has been shown to be inversely related to the risk of developing diabetes ^40^. These divergent outcomes across different populations underscore the impact of dietary habits and lifestyles on the study results. It is worth noting that the current study on Kurdish ethnicity in Iran represents the initial exploration of the relationship between the PDI and the risk of T2DM within this specific ethnic group, potentially providing unique insights into this association.

The outcomes of our study align with those of Kim et al., who demonstrated that individuals with higher mean PDI scores tend to exhibit increased daily intake of whole grains, fruits, vegetables, legumes, tea, and coffee. Conversely, they also tend to consume lower amounts of dairy, eggs, and meat. This similarity in dietary patterns strengthens the observations across different populations, highlighting the consistency of these associations between plant-based diets and specific food intake choices. ^40^. Indeed, the findings from our study align with prior research indicating the advantages of a plant-based diet in preventing diabetes. A cohort study conducted in China revealed that increased consumption of fruits, vegetables, nuts, and legumes and limited intake of red meat were associated with a reduced risk of T2DM ^31^.

Similarly, Lv et al. highlighted that adhering to a dietary pattern rich in fruits and vegetables while restricting red meat intake led to a substantial 26% reduction in the risk of developing T2DM ^32^. These consistent outcomes across diverse studies underscore the potential benefits of specific dietary choices in mitigating the risk of diabetes ^41^.

According to the Stockholm Diabetes Prevention Program, a clear inverse relationship was observed between high intake of fruits and vegetables and a decreased risk of T2DM^42^. Schwingshack et al. further supported this by demonstrating that increased consumption of fruits and vegetables correlates with a decreased incidence of T2DM^43^. Additionally, a prospective study among healthy females highlighted that higher intake of leafy green vegetables and fruits was associated with a decreased risk of developing T2DM ^44^. Meta-analyses also consistently underscored the risk reduction in T2DM among individuals consuming fruits, vegetables, soy products, and whole grains, contrasting with the increased risk linked to higher red meat consumption^43,45^. These collective findings emphasize the pivotal role of specific dietary patterns in lowering the risk of developing type 2 diabetes.

Certainly, the protective effect of a plant-based diet (PDI) against T2DM risk might involve various biological pathways. As phytochemicals are predominantly found in plant-based foods^46^, diets abundant in fruits and vegetables are strongly associated with a reduced risk of T2DM ^47^. A meta-analysis involving 18,164 patients with T2DM demonstrated that the overall intake of dietary flavonoids is linked to a decreased risk of developing T2DM ^48^. Additionally, another meta-analysis encompassing seven cohort studies revealed that individuals with higher flavonoid intake had an 11% lower likelihood of developing T2DM than did those with minimal flavonoid intake^49^. These findings underscore the potential role of phytochemicals and flavonoids present in plant-based foods in mitigating the risk of developing type 2 diabetes.

Certainly, the mechanisms underlying the potential benefits of a plant-based diet against T2DM encompass various aspects. The phytochemicals present in these diets exhibit antioxidant and anti-inflammatory properties, aiding in safeguarding pancreatic beta-cells from hyperglycemia and oxidative stress. Moreover, they support enhanced glucose absorption via insulin-dependent pathways, thereby improving insulin sensitivity and potentially preventing insulin resistance^50,51^. These compounds also have an impact on energy balance and regulate lipid and carbohydrate metabolism. Additionally, a plant-based diet is associated with improved intestinal microbiome health, increased dietary fibre content, and increased levels of unsaturated fatty acids, as well as decreased levels of saturated fatty acids, cholesterol, and animal protein^37^. For instance, an Umbrella Review analysing sixteen meta-analyses highlighted the significant association between higher fibre intake and a reduction in the relative risk of type 2 diabetes, fasting blood sugar concentration, and glycosylated hemoglobin levels among individuals with type 2 diabetes^52^. These factors collectively contribute to the potential protective effect of plant-based diets against the development and management of type 2 diabetes.

These findings are consistent with the proposed mechanisms. We observed a significant decrease in the number of individuals diagnosed with T2DM and a lower percentage of individuals consuming antidiabetic drugs as adherence to a plant-based diet increased. Additionally, our analysis revealed a decreasing trend in fasting blood sugar (FBS) levels with increasing plant-based diet index (PDI) tertiles. These results align with previous research by Lotfi et al., who demonstrated an inverse relationship between FBS concentration and greater adherence to a plant-based diet (OR = 0.42; 0.33–0.53)^53^. These findings reinforce the potential benefits of a plant-based diet in managing and possibly preventing type 2 diabetes, as indicated by the correlations observed in our study and in prior research.

The present study has several strengths, including a large sample size, adjustment for a wide range of potential confounders and the use of a validated questionnaire administered by trained specialists. However, there are limitations to consider. The cross-sectional study design prevented the establishment of causal relationships between the plant-based diet index (PDI) and type 2 diabetes mellitus (T2DM) incidence. Additionally, while the Food Frequency Questionnaire (FFQ), employed for determining PDI validity and reliability for evaluating plant foods, was used, it may not have captured the full dietary spectrum accurately. Despite controlling for various confounding factors, some potential confounders might not have been entirely accounted for in the study ^54^.

## Conclusion

Taken together, the findings of this study suggest that increased adherence to a plant-based diet comprising whole grains, fruits, vegetables, nuts, legumes, vegetable oils, and tea/coffee could lower the risk of developing type 2 diabetes mellitus (T2DM). However, further research is essential to validate and strengthen these results. This study underscores the importance of considering such dietary patterns to potentially mitigate the risk of T2DM, but additional investigations would be valuable to confirm and expand upon these findings.

## Data Availability

The data sets generated during this study are available from the corresponding author upon reasonable request via email.

## Abbreviations

BMI: Body mass index
CI: Confidence interval
CVDs: Cardiovascular diseases
DASH: Dietary Approaches to Stop Hypertension
FBS: Fasting blood sugar
FFQ: Food Frequency Questionnaire
GDM: Gestational diabetes mellitus
hPDI: Healthy plant-based diet index
IDF: International Diabetes Federation
PDI: Plant-based diet index
RaNCD: Ravansar Non-Communicable Disease
SES: Socioeconomic status
T: Tertiles
T2DM: Type 2 diabetes mellitus
VFA: Visceral fat area
WC: Waist circumference
